# The Malignant Imposter: Cystic Renal Oncocytoma Challenging Bosniak Criteria

**DOI:** 10.7759/cureus.104622

**Published:** 2026-03-03

**Authors:** Arjun N, Naveen Suraj, Dinesh Anne, Bhavya Shetty, Subhodaya R

**Affiliations:** 1 Department of Urology, Sapthagiri Institute of Medical Sciences and Research, Bengaluru, IND

**Keywords:** bosniak classification, cd117, chromophobe rcc, nephrectomy, renal oncocytoma

## Abstract

Renal cystic lesions are frequently encountered in clinical practice. While simple cysts are benign, complex cysts with solid components (e.g., Bosniak III/IV) often raise concerns regarding malignancy. Rarely, benign renal tumors such as oncocytoma may present with cystic changes, posing a significant diagnostic challenge.

A 70-year-old male was found to have a right renal cyst on abdominal imaging conducted for right upper quadrant fullness. Contrast-enhanced CT tomography of the abdomen revealed a 6.8 x 6.6 x 6.4 cm exophytic hypodense cystic lesion with an internal soft tissue component measuring 13 mm in thickness, which exhibited significant enhancement after contrast administration. Due to the suspicion of cancer, a right partial nephrectomy was performed. The patient had a smooth postoperative recovery. Histopathological analysis confirmed the lesion as a renal oncocytoma with cystic alterations. Immunohistochemistry tests were negative for CK7, confirming the diagnosis of renal oncocytoma. This case highlights the importance of histopathological and immunohistochemical assessments and emphasizes the need to prevent overtreatment of patients.

## Introduction

Renal cysts are common findings on abdominal imaging, with simple cysts being benign but complex cysts (Bosniak III/IV) raising malignancy concerns due to solid enhancing components [1]. The Bosniak classification guides management [2]; categories III and IV typically require surgery as they predict ~55-92% cancer risk [3].

Renal oncocytoma is a benign epithelial tumor accounting for approximately 3-7% of surgically excised renal neoplasms and typically presents as a solid renal mass, often with a central stellate scar [1]. Rarely (<1%), oncocytomas may demonstrate cystic or multicystic morphology, posing a significant diagnostic challenge due to radiologic overlap with cystic renal cell carcinoma [3-5]. We report a Bosniak IV cystic oncocytoma confirmed by CK7-/CD117- immunohistochemistry, highlighting management pitfalls.

## Case presentation

**Figure 1:**
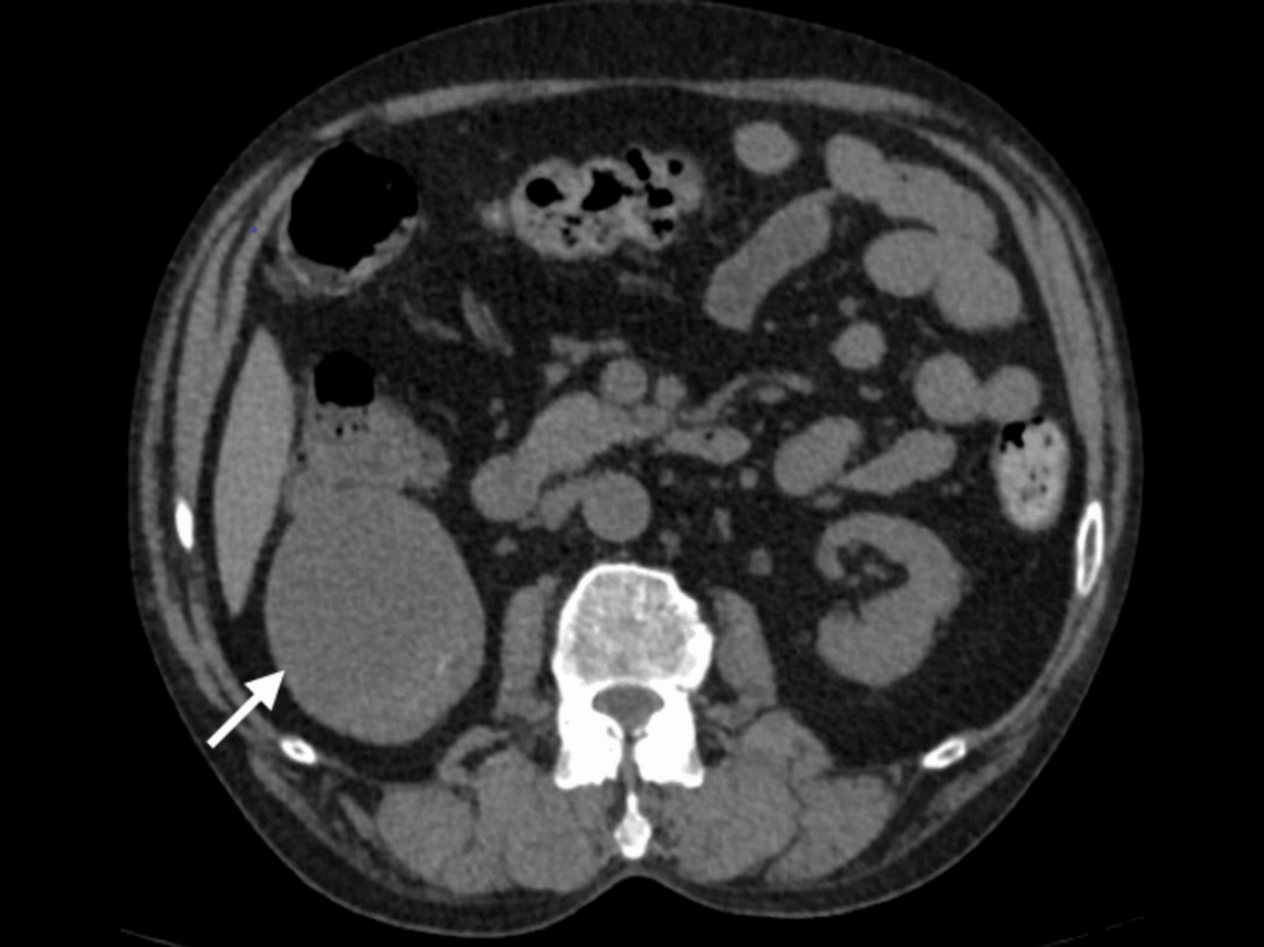
Axial contrast-enhanced CT demonstrates a well-defined cystic lesion with a peripheral location and homogeneous mural nodule enhancement (35 HU), and no perinephric fat stranding or lymphadenopathy.

**Figure 2:**
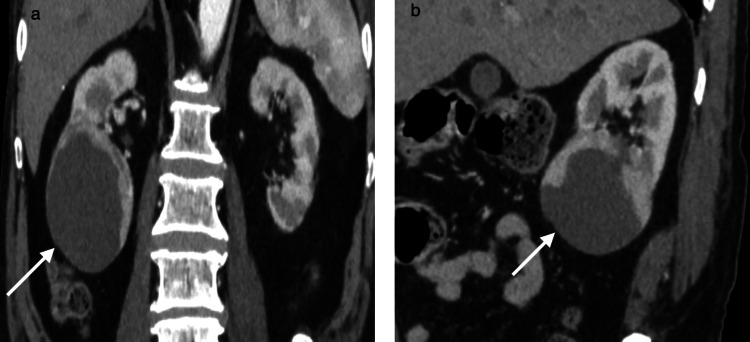
(a) Coronal contrast-enhanced CT (CECT) (corticomedullary phase) shows a 6.8 × 6.6 × 6.4 cm exophytic cystic lesion from the right lower pole abutting the inferior calyx. A 13 mm mural nodule enhances from 12 HU (pre) to 35 HU (post), consistent with Bosniak IV. No septations were noted. (b) Sagittal CECT confirms an exophytic lesion with an enhancing mural nodule (white arrow, 13 mm, 35 HU post-contrast). The lesion abuts the inferior polar artery without invasion.

A 70-year-old male patient with a history of diabetes and hypertension presented to the outpatient department with a history of fullness in the right upper quadrant for the past few months. No palpable mass was observed on examination. The patient underwent a contrast-enhanced CT scan, which revealed an exophytic cystic lesion arising from the right lower pole with an enhancing solid component abutting the inferior renal calyx and inferior polar artery (Figures 1, 2), enhancing from 12 HU (pre-contrast) to 35 HU (post-contrast). No septations were identified, and no regional lymphadenopathy was observed. The lesion was classified as Bosniak IV per the 2019 criteria [2]. Laboratory investigations revealed serum creatinine of 1.1 mg/dL (estimated glomerular filtration rate (eGFR) = 78 mL/min/1.73 m²), normal complete blood count (hemoglobin = 13.2 g/dL), no hematuria on urinalysis, and glycosylated hemoglobin (HbA1c) of 7.2%. After obtaining informed consent, a right open partial nephrectomy was performed, and the excised mass measured 8 x 6 cm on gross examination post fixation (preoperative CT: 6.8 x 6.6 x 6.4 cm) and was subjected to histopathological examination. The postoperative course was uneventful, and the patient was discharged on the fourth postoperative day, showing promising signs of recovery. The patient was followed for six months postoperatively. Follow-up included periodic clinical evaluation, serum creatinine and eGFR monitoring, ultrasonography at three months, and contrast-enhanced CT at six months. No evidence of recurrence was detected, and renal function remained stable throughout the follow-up period.

**Figure 3:**
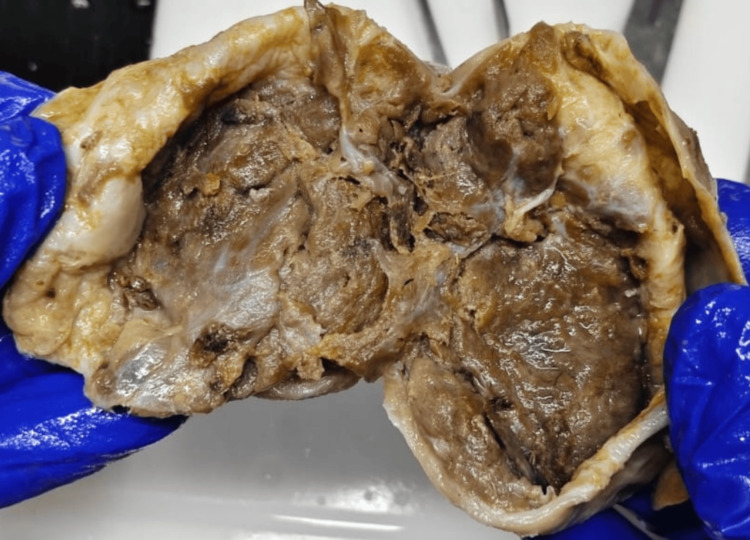
Gross pathology of renal oncocytoma with cystic changes. Macroscopic characteristics: the cyst wall shows an irregular mahogany brown lobulated lesion.

**Figure 4:**
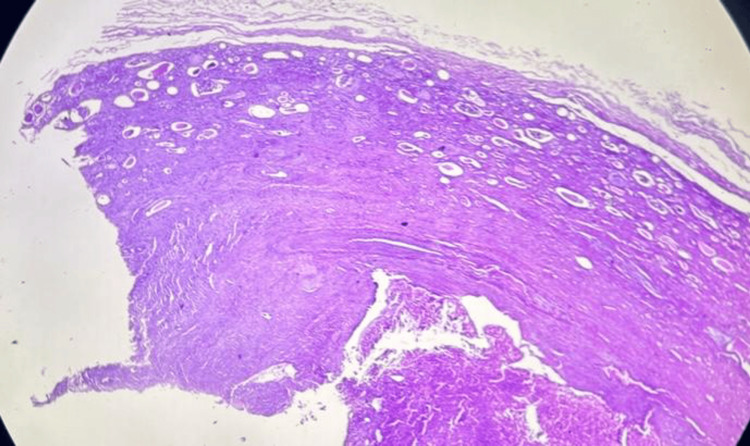
Histological features of cystic renal oncocytoma showing a thick fibrous wall with entrapped renal tubules containing proteinaceous cast, with occasional glomeruli.

**Figure 5:**
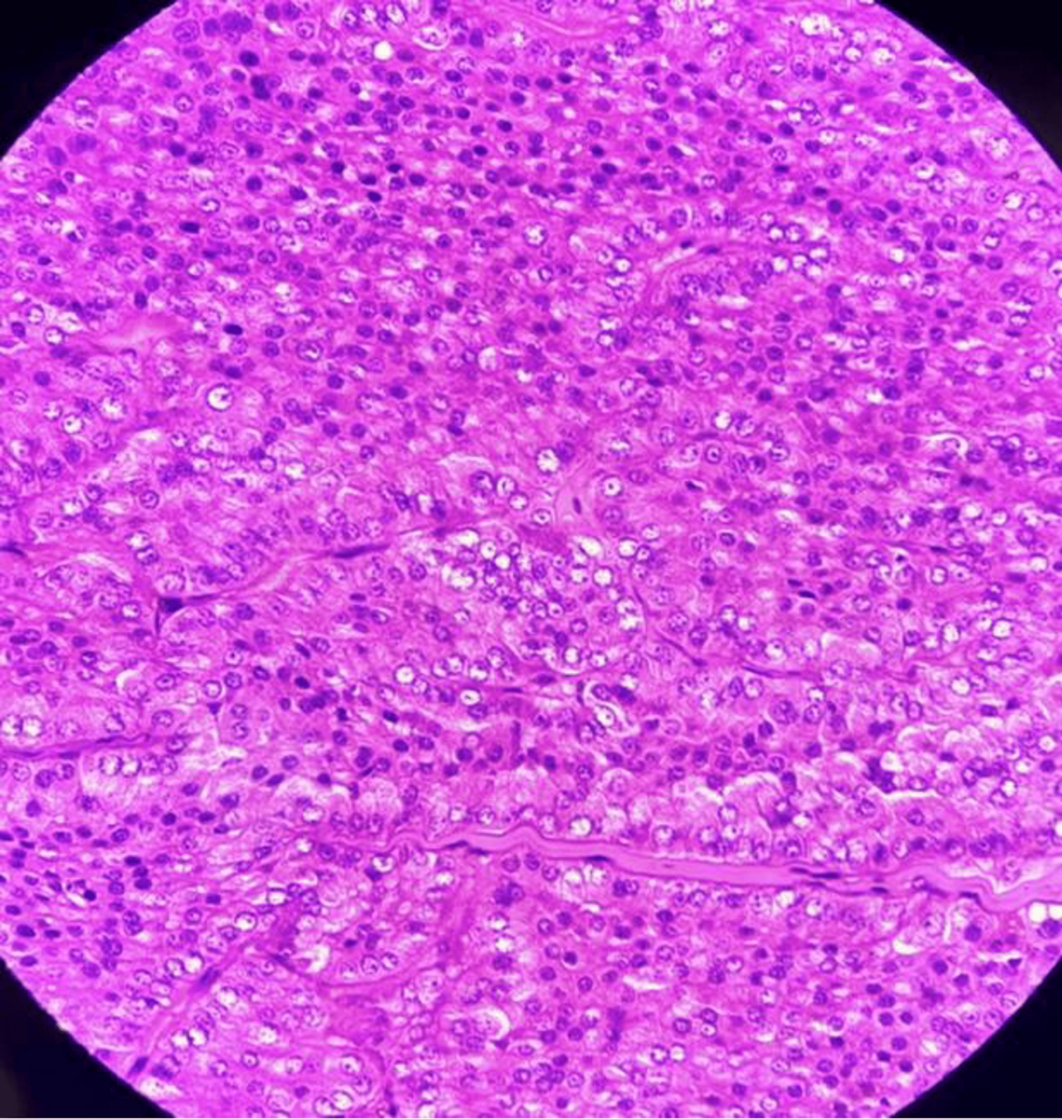
Histological features of cystic renal oncocytoma showing cells arranged in nests, groups, and an alveolar pattern separated by thin fibrous septae.

**Figure 6:**
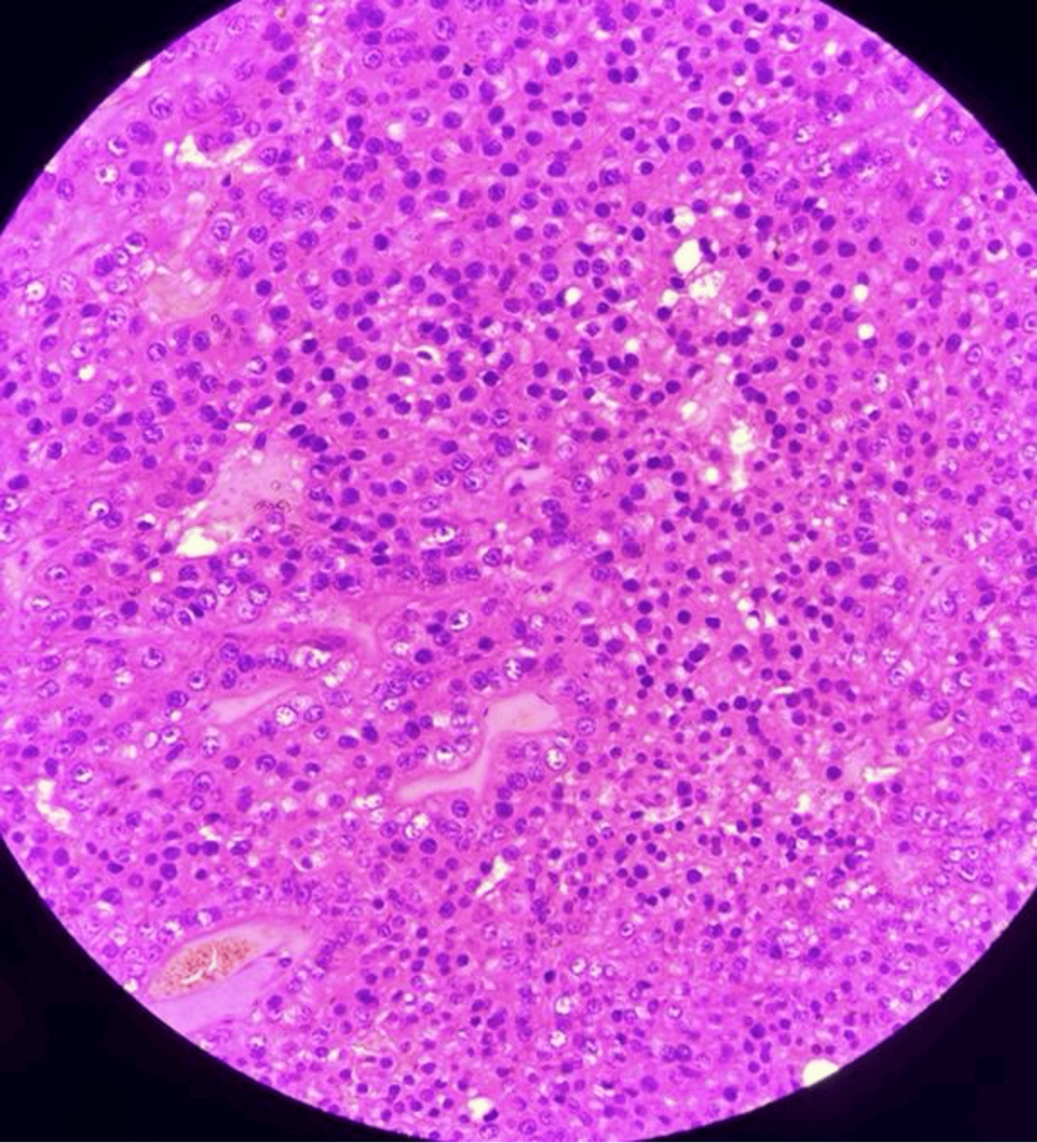
Histological features of cystic renal oncocytoma showing large round cells with dense eosinophilic granular cytoplasm (oncocytes) and round regular nuclei with even chromatin.

Gross examination of the excised specimen revealed a cystic lesion with an irregular mahogany-brown lobulated area along the cyst wall (Figure 3). Histopathological examination showed a thick fibrous cyst wall with entrapped renal tubules and occasional glomeruli (Figure 4). Tumor cells were arranged in nests, groups, and alveolar patterns separated by thin fibrous septae (Figure 5). Higher magnification revealed large polygonal oncocytes with abundant eosinophilic granular cytoplasm and round, regular nuclei (Figure 6). Immunohistochemistry showed tumor cells negative for CK7 and CD117, ruling out chromophobe renal cell carcinoma (Table 1).

**Table 1:** Immunohistochemical markers. RCC: renal cell carcinoma.

Marker	Result	Interpretation
CK7	Negative	Favors oncocytoma vs. chromophobe RCC
CD117	Negative	Favors oncocytoma vs. chromophobe RCC

## Discussion

Renal oncocytoma is a benign renal epithelial neoplasm originating from intercalated cells of the collecting duct and is characterized by a mahogany-brown gross appearance and abundant mitochondria-rich eosinophilic cytoplasm [1]. Although most oncocytomas are solid, cystic and multicystic variants are distinctly uncommon. Multicystic and multilocular oncocytomas have been reported in limited pathological series and isolated case reports [3,4,6]. Central cystic degeneration within small oncocytomas represents another rare morphologic presentation [5].

Radiologically, cystic oncocytomas may fulfill Bosniak III or IV criteria due to enhancing septations or mural nodules, resulting in a strong presumption of malignancy and surgical intervention. The imaging spectrum of oncocytoma shows considerable overlap with cystic renal cell carcinoma, including papillary and tubulocystic subtypes, making reliable preoperative differentiation difficult [7,8]. In the present case, the lesion fulfilled Bosniak IV criteria due to the presence of a contrast-enhancing mural nodule. According to the 2019 Bosniak update, enhancing nodularity within a cystic lesion confers a high likelihood of malignancy, often exceeding 80-90%, thereby warranting surgical intervention [2]. However, cystic renal oncocytomas may demonstrate true enhancement of mural nodules or septations, mimicking cystic renal cell carcinoma. The enhancement observed in oncocytomas reflects their rich mitochondrial content and vascular stroma rather than malignant behavior. This radiologic-pathologic discordance represents a key diagnostic challenge and underscores the limitation of imaging-based risk stratification alone.

Cystic alterations in oncocytoma, though uncommon (<5% of cases), typically stem from degenerative changes secondary to the tumor’s metabolic demands: abundant mitochondria impair vascular supply, promoting central ischemia, hemorrhage, and cyst formation with entrapped tubules (Figures 4, 5). Our case showed a thick fibrous wall with proteinaceous casts, reflecting this process, challenging Bosniak criteria preoperatively. Rare collision tumors, such as papillary renal cell carcinoma arising within a renal oncocytoma, have also been described and further complicate radiologic and pathologic interpretation [9].

Histologically, oncocytomas are composed of large polygonal cells arranged in nests, sheets, or alveolar patterns with abundant eosinophilic granular cytoplasm. Rare morphologic variants, including the small-cell variant, may closely mimic malignant renal neoplasms and represent a diagnostic pitfall [10]. Large clinicopathologic studies with long-term follow-up have consistently demonstrated the benign behavior of renal oncocytoma, even in tumors with atypical features [11]. Immunohistochemistry is critical for distinguishing oncocytoma from chromophobe renal cell carcinoma and other mimickers. Ancillary techniques, including colloidal iron staining and immunohistochemical marker panels, have been shown to improve diagnostic accuracy in challenging cases and help prevent overtreatment [12].

Although an MRI was not performed in this case, it may provide additional characterization in complex cystic lesions by better delineating septations and assessing enhancement patterns. However, even with advanced imaging modalities, reliable preoperative differentiation between cystic oncocytoma and cystic renal cell carcinoma remains challenging. Therefore, histopathological confirmation continues to be the gold standard for diagnosis.

## Conclusions

Cystic renal oncocytoma presents a rare diagnostic challenge owing to its close radiological resemblance to cystic renal cell carcinoma, particularly when categorized as Bosniak IV. This case highlights the limitations of imaging alone in characterizing complex cystic renal masses and emphasizes the importance of histopathology and immunohistochemistry for definitive diagnosis. CK7 and CD117 negativity supports the diagnosis, which is positive in many cases of chromophobe RCC. Nephron-sparing surgery remains the treatment of choice for lesions whenever feasible, offering curative outcomes with preservation of renal function. Awareness of this rare cystic variant is essential to avoid overtreatment and guide appropriate patient counselling and management. The absence of recurrence and stable renal function at six-month follow-up further reinforces the benign nature and excellent prognosis of cystic renal oncocytoma after complete resection.

## References

[1] Perez-Ordonez B, Hamed G, Campbell S, Erlandson RA, Russo P, Gaudin PB, Reuter VE (1997). Renal oncocytoma: a clinicopathologic study of 70 cases. Am J Surg Pathol.

[2] Silverman SG, Pedrosa I, Ellis JH (2019). Bosniak classification of cystic renal masses, version 2019: an update proposal and needs assessment. Radiology.

[3] Ogden BW, Beckman EN, Rodriguez FH Jr (1987). Multicystic renal oncocytoma. Arch Pathol Lab Med.

[4] Schmidt PR, Böck D, Gasser G, Redtenbacher S (1991). Multicystic renal oncocytoma. (Article in German). Urologe A.

[5] Kodama K, Nagano K, Akimoto M, Suzuki S (2004). Small renal oncocytoma with central cystic degeneration. Int J Urol.

[6] Leroy X, Aubert S, Lemaitre L, Haffner J, Biserte J, Gosselin B (2006). Multilocular cystic renal oncocytoma. J Clin Pathol.

[7] Ishigami K, Jones AR, Dahmoush L, Leite LV, Pakalniskis MG, Barloon TJ (2015). Imaging spectrum of renal oncocytomas: a pictorial review with pathologic correlation. Insights Imaging.

[8] Skenderi F, Ulamec M, Vranic S (2016). Cystic renal oncocytoma and tubulocystic renal cell carcinoma: morphologic and immunohistochemical comparative study. Appl Immunohistochem Mol Morphol.

[9] Rowsell C, Fleshner N, Marrano P, Squire J, Evans A (2007). Papillary renal cell carcinoma within a renal oncocytoma: case report of an incidental finding of a tumour within a tumour. J Clin Pathol.

[10] Hes O, Michal M, Boudova L, Mukensnabl P, Kinkor Z, Miculka P (2001). Small cell variant of renal oncocytoma—a rare and misleading type of benign renal tumor. Int J Surg Pathol.

[11] Dvorakova M, Dhir R, Bastacky SI (2010). Renal oncocytoma: a comparative clinicopathologic study and fluorescent in-situ hybridization analysis of 73 cases with long-term follow-up. Diagn Pathol.

[12] Tickoo SK, Amin MB, Zarbo RJ (1998). Colloidal iron staining in renal epithelial neoplasms, including chromophobe renal cell carcinoma: emphasis on technique and patterns of staining. Am J Surg Pathol.

